# H19 Overexpression Improved Efficacy of Mesenchymal Stem Cells in Ulcerative Colitis by Modulating the miR-141/ICAM-1 and miR-139/CXCR4 Axes

**DOI:** 10.1155/2021/7107705

**Published:** 2021-09-29

**Authors:** Ming-li Zhao, Tao Chen, Teng-hui Zhang, Feng Tian, Xiao Wan

**Affiliations:** ^1^Department of General Surgery & Guangdong Provincial Key Laboratory of Precision Medicine for Gastrointestinal Tumor, Nanfang Hospital, The First School of Clinical Medicine, Southern Medical University, Guangzhou, Guangdong 510515, China; ^2^Department of General Surgery, Jinling Hospital, School of Medicine, Nanjing University, Nanjing, Jiangsu 210002, China; ^3^Department of Gastrointestinal Surgery, Provincial Hospital Affiliated to Shandong University, Jinan, Shandong 250012, China; ^4^Department of General Surgery, The First Affiliated Hospital of USTC, Division of Life Sciences and Medicine, University of Science and Technology of China, Hefei, Anhui 230001, China

## Abstract

Overexpression of C-X-C motif chemokine receptor 4 (CXCR4) and intercellular cell adhesion molecule-1 (ICAM-1) may promote homing of mesenchymal stem cells (MSC). In this study, we treated ulcerative colitis animals with MSC preconditioned with or without H19 and compared the therapeutic effect of MSC and MSC-H19. We evaluated the regulatory relationship of H19 vs. miR-141/miR-139 and miR-141/miR-139 vs. ICAM-1/CXCR4. We established an ulcerative colitis mouse model to assess the effect of MSC and MSC-H19. H19 was found to bind to miR-141 and miR-139. The activity of H19 was strongly decreased in cells c-transfected with miR-141/miR-139 and WT H19. ICAM-1 was confirmed to be targeted by miR-141 and CXCR4 was targeted by miR-139. The H19 expression showed a negative regulatory relationship with the miR-141 and miR-139 expression but a positive regulatory relationship with the ICAM-1 and CXCR4 expression. In summary, the overexpression of H19 in MSC downregulated miR-139 and miR-141, thus increasing the activity of their targets ICAM-1 and CXCR4, respectively, to exhibit therapeutic effects in ulcerative colitis.

## 1. Introduction

As a condition of the gastrointestinal tract system, inflammatory bowel disease (IBD) manifests as ulcerative colitis, Crohn's disease, and persistent inflammation in the stomach. The etiology of IBD remains unclear; although, studies on IBD pathogenesis have shown that the breakdown of the immune system in the digestive tract, including an imbalance in intestinal microbiota, is crucial for the onset of this condition [1, 2].

Mesenchymal stem cells (MSC) are present in many body organs, such as dental pulp, bone marrow, fat, and muscles; furthermore, MSC are linked to the microvasculature in the entire body as pericytes. These cells may differentiate into different types of mesenchymal cells [3]. MSC produce cytokines and alter the microenvironment required for cell regrowth. MSC possess strong immunomodulatory properties by reducing the proliferation of inflammatory cells as well as the synthesis of cytokines [4]. MSC are presently utilized for the treatment of inflammatory conditions, such as myocarditis, sclerosis, and IBD [5, 6]. While the functions of MSC remain elusive, MSC are used for IBD therapy and have displayed appealing outcomes in animals [7–9].

Noncoding RNA (ncRNA) has been shown to possess significant properties in the regulation of gene functions [10]. NcRNAs have been shown to become significant regulatory factors in the onset, progression, and prognosis of medical conditions [11]. NcRNAs include the extensively studied miRNAs, as well as the lncRNAs, which is one type of ncRNA recently recognized for their importance. However, although miRNAs have been intensively examined for their functionalities, lncRNAs also comprise a new and possibly crucial class of ncRNAs [12]. LncRNA H19 is located close to the telomeric region of chromosome 11p15.5 [13], and it was reported to be highly expressed in the developing embryo while being significantly downregulated in neonates [14]. Moreover, H19 has been described as an oncogene in several diseases such as bladder cancer, colorectal cancer, or breast cancer [15–17]. Apart from this, H19 silences target genes by upregulating the miR-139 expression. In one study, H19 was significantly upregulated under hypoxic conditions, indicating that H19 participates in cell injury induced by hypoxia [18]. And a previous study showed the that miR-139 expression was downregulated in nerve cells and rodent brains under hypoxia [19]. Moreover, H19 was demonstrated to compete with miR-141 and influence the expression of miR-141, as well as the ZEB1 which is the target gene of miR-141, in gastric cancer [20]. By making use of a bioinformatic approach, H19 was shown to bind to miR-139 and miR-141. Therefore, it was supposed that there may be interactions between miR-139/miR-141 and H19 [20, 21].

Bioinformatic results and dual-luciferase assays have suggested that CXCR4 is a direct miR-139 target. Functionally, the suppressive effects of miR-139 on the proliferation of T-ALL cells were reversed by CXCR4 transfection, while the miR-139 overexpression substantially lowered the malignancy of T-ALL cells, and people with very high expression of miR-139 and reduced CXCR4 expression showed greater five-year survival than people with reduced miR-139 expression [22]. Also, the homing and migratory capacity of umbilical cord-derived MSC via regulating CXCR4 could lead to the boosting of protection against liver ischemia/reperfusion injury by rapamycin-preconditioned umbilical cord-derived MSC [23], and by promoting the expression of CXCR4, HIV glycoprotein gp120 has also been found to enhance the migration of MSC [24]. Also, bioinformatic results and dual-luciferase assays have suggested ICAM-1 as a direct target of miR-141. ICAM-1 has been shown to interact with target cells during cytotoxic T cell and natural killer cell-mediated damage [25], and ICAM-1 has also been shown to prevent and reverse dextran sulfate sodium-induced colitis in mice [26].

Moreover, It has been shown that the overexpression of CXCR4 and ICAM-1 may promote the homing of MSC in the treatment of IBD [27, 28]. Furthermore, we suspected that the overexpression of H19 may enhance the expression of CXCR4 and ICAM-1, possibly by the findings that H19 could sponge the expression of miR-139 and miR-141 expression [21]. In this study, we treated ulcerative colitis animals with MSC preconditioned with or without H19 and compared the therapeutic effect of MSC and MSC-H19 in the treatment of ulcerative colitis.

## 2. Materials and Methods

### 2.1. Mice

All female C57BL/6 mice (approximately 8 weeks, body weight approximately 20 g) were obtained from the institutional animal center. Mice were fed a pellet-based diet and drinking water. The study protocol was approved by the institutional ethical committee.

### 2.2. Preparation of MSC

Bilateral tibias and femurs were isolated from four-week-old female C57BL/6 mice, and bone marrow was rinsed out of the marrow cavity with DMEM (Gibco, New York, NY, USA) and filtered using a cell strainer (70 *μ*m, BD Biosciences, Franklin Lakes, NJ, USA) prior to spinning for 5 mins at 600 g. The cell pellets were then resuspended in the same medium at 1 × 10^6^ cells/mL. H19- and CXCR4-overexpressing MSC were produced using lentiviral transduction (Thermo Fisher, USA) with pWSLV-07-EF1a-Puro-GFP vectors. The correctness of each plasmid was validated through sequencing. 293 T cells (ATCC, Manassas, VA) were cotransfected with the pWSLV-07-EF1a-H19-Puro-GFP and pWSLV-07-EF1a-Puro-GFP plasmids using Lipofectamine 3000 (Invitrogen, Carlsbad, CA).

### 2.3. Animal Model

Colitis was triggered by administration of 36–50 kDa DSS (MP Biomedical, Santa Ana, CA). The mice were fed 5% DSS in sterile water for 1 week and then fed regular water for another 14 days (three cycles). The animals were evaluated every day to record food uptake and body weight (body weight change was calculated using the weight on day 0 as a reference). Bodyweight data were collected every 3 days to calculate body weight change (%) (weight at day *X*/weight at day 0) × 100), and stool consistency and rectal bleeding occurrence were recorded. Thirty-two C57BL/6 mice were randomly divided into four groups (eight animals/group): a SHAM group (mice treated with PBS), animal model group (mice treated with DSS), and two treatment groups (mice treated with DSS + MSC and mice treated with DSS + MSC-H19). For the two treatment groups, mice were treated with MSC or MSC-H19 (3 × 10^6^ cells/mL) via tail vein injection on days 4, 14, and 24. The animal model establishment was accomplished following previous published protocols [29]. Around 5% of the mice died during our experiment. At the end of the experiments (12 weeks in total), the animals in each group were killed to isolate colons for functional analysis.

### 2.4. Real-Time PCR

A miRNeasy Mini kit (Qiagen, Valencia, CA) was used with a ReverTra PCR assay kit (Toyobo, Osaka, Japan) and SYBR Green mix (Toyobo, Osaka, Japan) to measure H19, miR-141, CXCR4, ICAM-1, and miR-139 expression with the 2^−*ΔΔ*Ct^ method. U6 and *β*-actin were used for control. The primer sets for H19 were 5′-CTTCTTTAAGTCCGTCTCGTTC-3′ (forward) and 5′-GAGGCAGGTAGTGTAGTGGTTC-3′ (reverse). The primer sets for miR-141 were 5′-TCTTCCAGTGCAGTGTTGG-3′ (forward) and 5′-GAACATGTCTGCGTATCTC-3′ (reverse). The primer sets for miR-139 were 5′-CTACAGTGCACGTGTCTC-3′ (forward) and 5′-GAACATGTCTGCGTATCTC-3′ (reverse). The primer sets for CXCR4 mRNA were 5′-GACTGGCATAGTCGGCAATGGA-3′ (forward) and 5′-CAAAGAGGAGGTCAGCCACTGA-3′ (reverse). The primer sets for ICAM-1 mRNA were 5′-AAACCAGACCCTGGAACTGCAC-3′ (forward) and 5′-GCCTGGCATTTCAGAGTCTGCTo-3′ (reverse).

### 2.5. Cell Experiments

MSC were divided into 2 groups: (1) a pcDNA group (MSC transfected with pcDNA) and (2) a pcDNA-H19 group (MSC transfected with pcDNA-19). The cells were cultured in DMEM (Gibco, Waltham, MA) containing 100 U/mL pen-strep and 10% FBS. Cells were passaged using 0.25% trypsin EDTA (Gibco, Thermo Fisher Scientific, Waltham, MA) every week. Similarly, MSC were divided into 2 groups: (1) a scramble control group (MSC transfected with control siRNA) and (2) an H19 siRNA group (MSC transfected with H19 siRNA). The primer sets for H19 siRNA were 5′-AACCCGUAGAUCCGAUCUUGUG-3′ (forward) and 5′-CAAGAUCGGAUCUACGGGUUU-3′ (reverse). The primer sets for control siRNA were 5′-UUCUCCGAACGUGUCACGUTT-3′ (forward) and 5′-ACGUGACACGUUCGGAGAATT-3′ (reverse). The transfection of MSC was performed with Lipofectamine 3000, and cells were collected 48 h after transfection for subsequent assays.

### 2.6. Cell Invasion Assay

Transwell assays (Corning, Corning, NY) were performed using an 8 *μ*m membrane with a Matrigel coating (BD Biosciences, San Jose, CA).

### 2.7. Luciferase Assay

According to previous publications, H19 was predicted to bind to miR-141 [20] and miR-139 [21]. To confirm the binding of H19 to miR-141 and miR-139, a luciferase assay was performed by cotransfecting cells with scrambled miRNA control + WT H19, miR-141/miR-139 + WT H19, scrambled miRNA control + MUT H19, and miR-141/miR-139 + MUT H19. In brief, fragments of miR-141 and miR-139 containing miR-141 and miR-139 binding sites were cloned into the pcDNA vector (Promega, Madison, WI) downstream of the luciferase gene to generate wild-type H19 plasmids for miR-141 and miR-139 binding, respectively. At the same time, site-directed mutagenesis was performed at the miR-141 and miR-139 binding sites of H19 to generate mutant H19 plasmids. In the next step, MSC were cotransfected with wild-type/mutant H19 and miR-141 or miR-139 using Lipofectamine 3000. The luciferase activity was tested 48 h later using a dual-Luciferase assay (Promega, Madison, WI).

Moreover, by searching public databases (http://mirtarbase.mbc.nctu.edu.tw, http://www.mirdb.org), ICAM1 and CXCR4 were shown to, respectively, bind to miR-141 and miR-139. In this study, fragments of ICAM-1 and CXCR4 containing binding sites for miR-141 and miR-139, respectively, were cloned into pcDNA vectors downstream of the luciferase gene to generate wild-type ICAM-1 and CXCR4 plasmids for miR-141 and miR-139 binding, respectively. At the same time, site-directed mutagenesis was performed at the miR-141 and miR-139 binding sites of ICAM-1 and CXCR4 to generate mutant-type ICAM-1 and CXCR4 plasmids for miR-141 and miR-139 binding, respectively. In the next step, MSC were cotransfected with wild-type/mutant-type ICAM-1 and CXCR4 plasmids and miR-141 or miR-139 using Lipofectamine 3000. Universally, transfection was performed following the general protocol: the concentration of plasmid-based constructs was 0.2 *μ*g, and the concentration of miRNA mimics was 30 nM. The constructs and mimics were separately dissolved in PBS and added to the medium in a dish before shaking to mix. The luciferase activity of the cells was assayed 48 h posttransfection by using a Dual-Luciferase Reporter assay kit.

### 2.8. Western Blotting

Western blotting was done to assay the expression of ICAM-1 and CXCR4 proteins in samples using a conventional procedure. Anti-ICAM-1 and anti-CXCR4 primary antibodies were purchased from Abcam (Cambridge, MA).

### 2.9. MTT Assay

MSC proliferation was measured using an MTT assay kit (Beyotime Medical, Wuhan, China). Optical density was evaluated at 570 nm wavelength on a microplate reader (Bio-Rad 550, Bio-Rad Laboratories, Hercules, CA) in strict accordance with the manual provided by the machine manufacturer.

### 2.10. Transwell Assay

Transwells (8 *μ*m, Millipore, Billerica, MA) were used to examine the migration ability of MSC in a 48 h experiment.

### 2.11. Immunohistochemistry

Immunohistochemistry was carried out to determine the protein expression of ICAM-1 and CXCR4 in paraffin-embedded samples. After rehydration, the 4 *μ*m sliced tissue sample sections were blocked using 5% goat serum before they were incubated with anti-ICAM-1 (dilution: 1 : 1,000; Abcam, Cambridge, MA) and anti-CXCR4 primary antibodies (dilution: 1 : 1,000; Abcam, Cambridge, MA) at 4°C overnight. Then, the sections were incubated for twenty minutes at 37°C with biotinylated secondary antibodies (dilution: 1 : 2,000; Abcam), stained with 3,3′diaminobenzidine (DAB, Thermo Fisher Scientific, Waltham, MA), counterstained with hematoxylin, and observed under an Olympus light microscope (Olympus Corporation).

### 2.12. Histological Analysis

HE stained transverse sections from colon samples were sliced into 4 *μ*m sections, fixed at 4°C overnight in 4% (v/v) formaldehyde, and embedded in paraffin. The extent of colonic inflammatory damage was assessed blindly according to previous publications [30].

### 2.13. Serum Analysis

The levels of peripheral blood biomarkers, such as C-reactive protein (CRP), were evaluated with a biochemistry analyzer (TBA-120FR; Toshiba Medical System Co., Tochigi, Japan) according to the manufacturer's instructions.

## 3. Statistical Analysis

One-way ANOVA (post hoc test: Tukey's test) and Student's *t*-tests were performed using SPSS 21.0 when appropriate to compare the results for different groups. A *P* value of 0.05 was taken to judge statistical significance.

## 4. Results

### 4.1. Validation of the H19-miR-141-ICAM-1 and H19-miR-139-CXCR4 Axes

**Figure 1:**
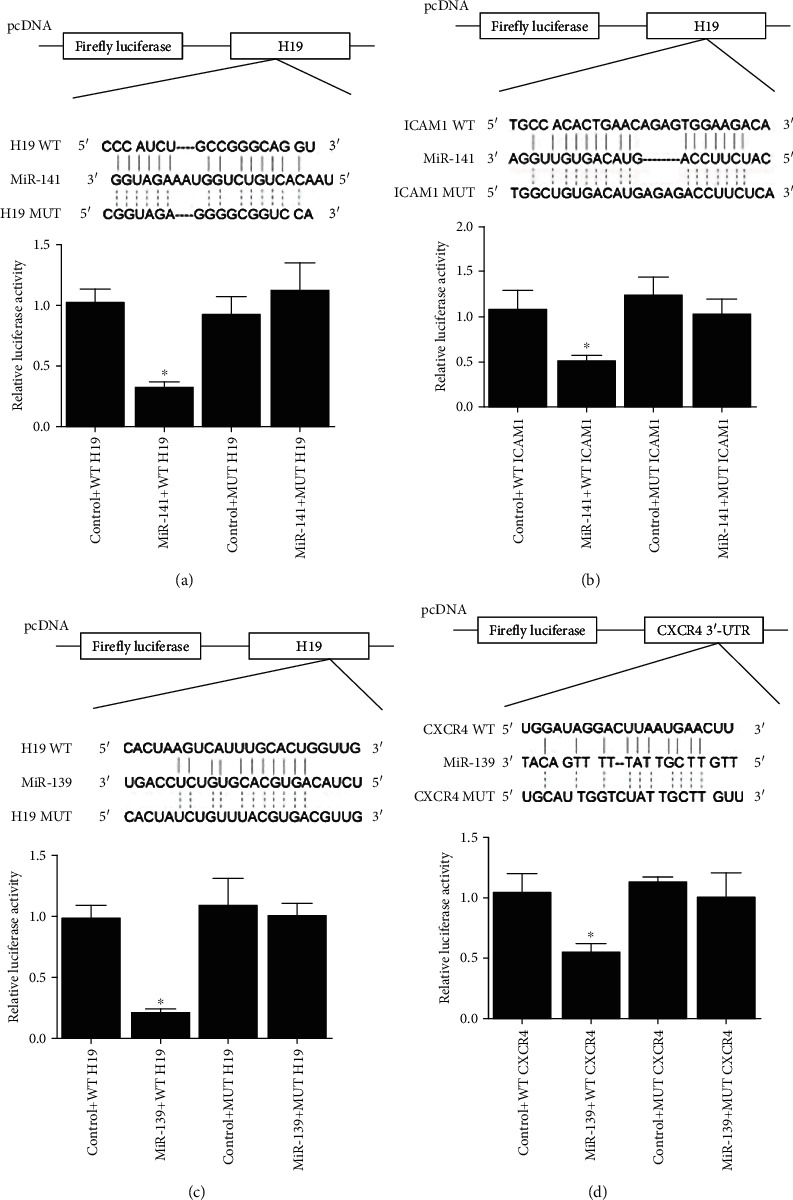
Luciferase assay of the H19-miR-141-ICAM-1 and H19-miR-139-CXCR4 axis. (a) Binding region between H19 and miR-141 was predicted, and the luciferase activity was the lowest in cells cotransfected with miR-141 and wild-type H19 (^∗^*P* value <0.05 vs. control + WT H19). (b) Binding region between miR-141 and ICAM-1 was predicted, and the luciferase activity was the lowest in cells cotransfected with miR-141 and wild-type ICAM-1 (^∗^*P* value <0.05 vs. control + WT ICAM1). (c) Binding region between H19 and miR-139 was predicted, and the luciferase activity was the lowest in cells cotransfected with miR-139 and wild-type H19 (^∗^*P* value <0.05 vs. control + WT ICAM1). (d) Binding region between miR-139 and CRCX4 was predicted, and the luciferase activity was the lowest in cells cotransfected with miR-139 and wild-type CRCX4 (^∗^*P* value <0.05 vs. control + WT CXCR4).

By searching public databases (http://mirtarbase.mbc.nctu.edu.tw and http://www.mirdb.org), H19 was predicted to bind to miR-141 (Figure 1(a)) and miR-139 (Figure 1(c)). We subsequently confirmed the binding of H19 to miR-141 and miR-139 by detecting the luciferase activity in cells cotransfected with control + WT/MUT H19 and in cells cotransfected with miR-141/miR-139 + WT/MUT H19. Accordingly, the results revealed that the luciferase activity was sharply decreased in cells cotransfected with WT H19 and miR-141 (Figure 1(a)) or WT H19 and miR-139 (Figure 1(c)). The downstream targets of miR-141 and miR-139 were further analyzed. ICAM-1 was predicted to be a downstream target of miR-141 (Figure 1(b)), and CXCR4 was predicted to be a downstream target of miR-139 (Figure 1(d)). Then, the luciferase activity was tested in cells cotransfected with control + WT/MUT ICAM-1 compared with cells cotransfected with miR-141 + WT/MUT ICAM-1 (Figure 1(b)) and in cells cotransfected with control + WT/MUT CXCR4 compared with cells cotransfected miR-139 + WT/MUT CXCR4 (Figure 1(d)). Accordingly, luciferase activity was significantly decreased in cells cotransfected with miR-141 and wild-type ICAM-1 (Figure 1(b)) and in cells cotransfected with CXCR4 and miR-139 (Figure 1(d)).

### 4.2. Regulatory Relationship among H19, miR-141/miR-139, and ICAM-1/CXCR4

**Figure 2:**
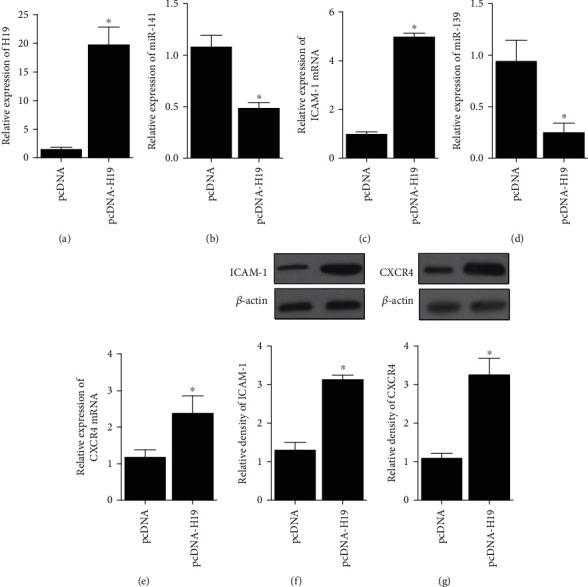
RNA and protein levels of miR-141, miR-139, ICAM-1, and CXCR4 in MSC was regulated by the transfection of H19 (^∗^*P* value <0.05 vs. pcDNA). (a) RNA level of H19 was boosted in MSC transfected with H19. (b) RNA level of miR-141 was decreased in MSC transfected with H19. (c) mRNA level of ICAM-1 was increased in MSC transfected with H19. (d) RNA level of miR-139 was decreased in MSC transfected with H19. (e) mRNA level of CXCR4 was increased in MSC transfected with H19. (f) protein level of ICAM-1 was higher in MSC transfected with H19. (g) Protein level of CXCR4 was higher in MSC transfected with H19.

**Figure 3:**
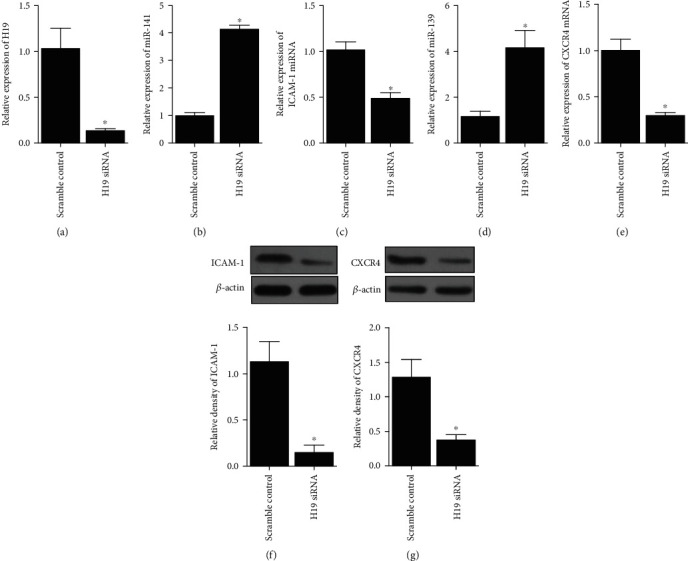
RNA and protein levels of miR-141, miR-139, ICAM-1, and CXCR4 in MSC transfected with scramble control or H19 siRNA (^∗^*P* value <0.05 vs. control). (a) RNA level of H19 was reduced by the transfection of H19 siRNA in MSC. (b) RNA level of miR-141 was promoted by the transfection of H19 siRNA in MSC. (c) mRNA level of ICAM-1 was inhibited by the transfection of H19 siRNA in MSC. (d) RNA level of miR-139 was promoted by the transfection of H19 siRNA in MSC. (e) mRNA level of CXCR4 was inhibited by the transfection of H19 siRNA in MSC. (f) Protein level of ICAM-1 was reduced with the presence of H19 siRNA in MSC. (g) Protein level of CXCR4 was reduced with the presence of H19 siRNA in MSC.

Mesenchymal stem cells (MSC) were transfected with pcDNA or pcDNA-H19, followed by detection of the expression of miR-141, ICAM-1 mRNA/protein, miR-139, and CXCR4 mRNA/protein. The evidently upregulated H19 expression validated the successful transfection of pcDNA-H19 (Figure 2(a)), and the miR-141 (Figure 2(b)) and miR-139 (Figure 2(d)) expression was decreased in pcDNA-H19 cells, while the ICAM-1 (Figures 2(c) and 2(f)) and CXCR4 (Figures 2(e) and 2(g)) expression was increased in pcDNA-H19 cells. In addition, cells transfected with H19 siRNA (Figure 3(a)) had higher levels of miR-141 (Figure 3(b)) and miR-139 (Figure 3(d)) but lower levels of ICAM-1 (Figures 3(c) and 3(f)) and CXCR4 (Figures 3(e) and 3(g)).

### 4.3. The Impacts of H19 on Cell Proliferation and Migration

**Figure 4:**
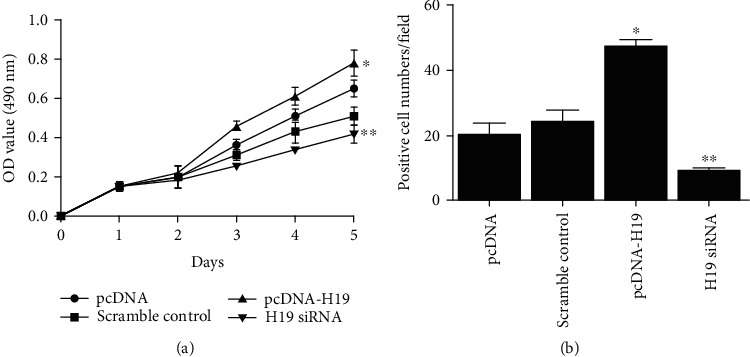
Proliferation and migration of MSC transfected with pcDNA, scramble control, pcDNA-H19, or H19 siRNA (^∗^*P* value <0.05 vs. pcDNA; ^∗∗^*P* value <0.05 vs. control). (a) MTT assay indicated that proliferation of MSC transfected with pcDNA-H19 was the highest among all cell groups. (b) Transwell assay indicated that migration of MSC transfected with pcDNA-H19 was the highest among all cell groups.

MSC were transfected with pcDNA, scramble control, pcDNA-H19, or H19 siRNA, and MTT and transwell assays were performed to detect cell proliferation and migration. MTT results showed that the proliferation rate of cells transfected with pcDNA-H19 was significantly higher than that of cells in other groups, while the proliferation rate of cells transfected with H19 siRNA was the lowest among all the cell groups (Figure 4(a)). Transwell assays revealed that higher expression of H19 had positive effects on cell migration, while downregulation of H19 inhibited cell migration (Figure 4(b)).

### 4.4. Impact of MSC and H19 on Ulcerative Colitis Mice

**Figure 5:**
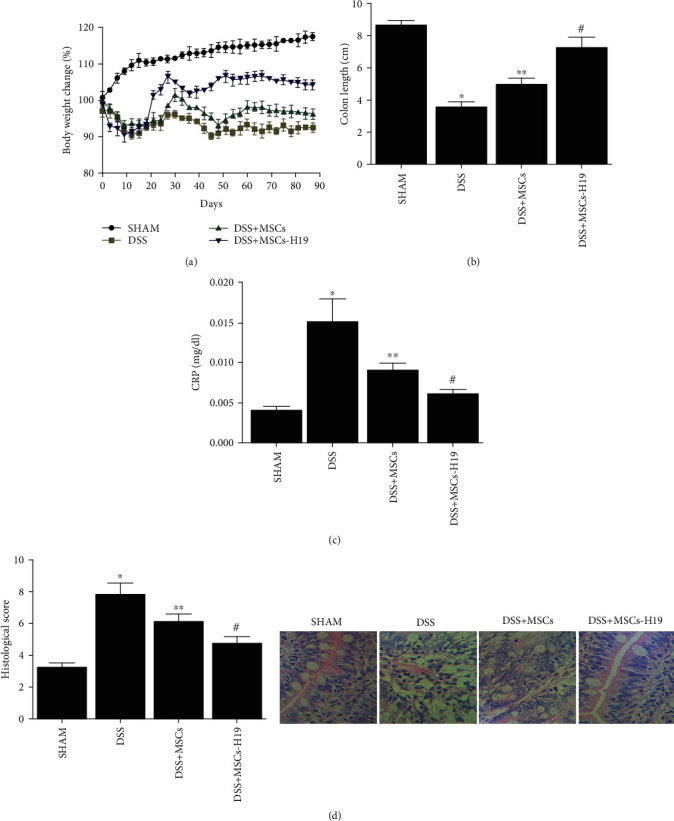
Effects of DSS, MSC, and MSC-H19 on mice weight and colon length. (a) Body weight loss of mice was highest in the DSS group, while MSC treated and MSC-H19 treated both obstructed this negative effect (body weight loss (%) = (weight at day *X*/weight at day 0) × 100). (b) Colon length of mice was shortest in the DSS group and longest in the SHAM group (^∗^*P* value <0.05 vs. control; ^∗∗^*P* value <0.05 vs. DSS; #*P* value <0.05 vs. DDS + MSC). (c) Histological inflammatory score of HE staining of mice was the highest in the DSS group and lowest in the SHAM group (^∗^*P* value <0.05 vs. control; ^∗∗^*P* value <0.05 vs. DSS; #*P* value <0.05 vs. DDS + MSC). (d) CRP level of mice was the highest in the DSS group and lowest in the SHAM group (^∗^*P* value <0.05 vs. control; ^∗∗^*P* value <0.05 vs. DSS; #*P* value <0.05 vs. DDS + MSC).

Mice were randomly divided into 4 groups: SHAM, DSS, DSS + MSC ,and DSS + MSC-H19, and body weight was measured every three days. Accordingly, at day 90, mice treated with DSS showed significant weight loss (Figure 5(a)) and shortened colon length (Figure 5(b)), and the effects of DSS were attenuated by MSC treatment and further alleviated by MSC-H19 treatment (Figures 5(a) and 5(b)). Additionally, the level of the peripheral blood biomarker CRP was the lowest in the SHAM group and highest in the DSS group (Figure 5(c)). As shown in Figure 5(d), HE staining indicated that the level of inflammation was evidently increased by DSS, and MSC treatment and MSC-H19 treatment partially restored the increased histological inflammatory score in DSS mice.

**Figure 6:**
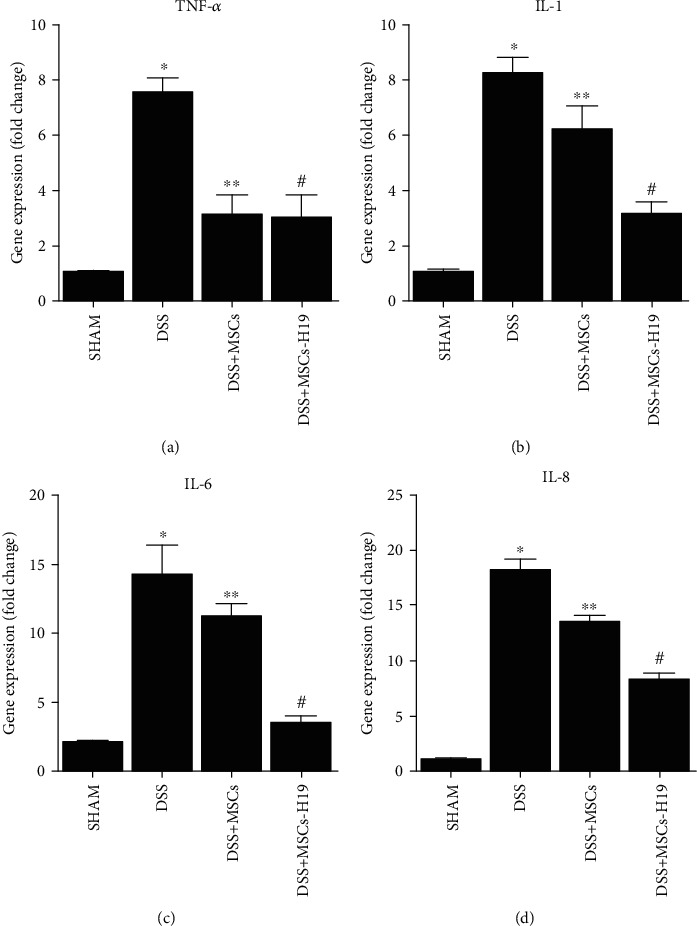
Effects of DSS, MSC, and MSC-H19 on the inflammatory response were measured by the detection of mRNA levels of TNF-*α*, IL-1, IL-6, and IL-8 (^∗^*P* value <0.05 vs. control; ^∗∗^*P* value <0.05 vs. DSS; #*P* value <0.05 vs. DDS + MSC). (a) Level of TNF-*α* mRNA in mice was the highest and lowest, respectively, in the DSS and SHAM groups. (b) Level of IL-1 mRNA in mice was the highest and lowest, respectively, in the DSS and SHAM groups. (c) Level of IL-6 mRNA in mice was the highest and lowest, respectively, in the DSS and SHAM groups. (d) Level of IL-8 mRNA in mice was the highest and lowest, respectively, in the DSS and SHAM groups.

Then, the levels of inflammatory cytokines (TNF-*α*, IL-1, IL-6, IL-8) were tested in the four groups. As expected, DSS treatment induced an inflammatory response, manifested by increased expression of TNF-*α* (Figure 6(a)), IL-1 (Figure 6(b)), IL-6 (Figure 6(c)), and IL-8 (Figure 6(d)). MSC injection reduced the effects of DSS, and MSC-H19 infusion further reduced the level of inflammatory responses.

### 4.5. Functions of MSC and H19 in Ulcerative Colitis Mice

**Figure 7:**
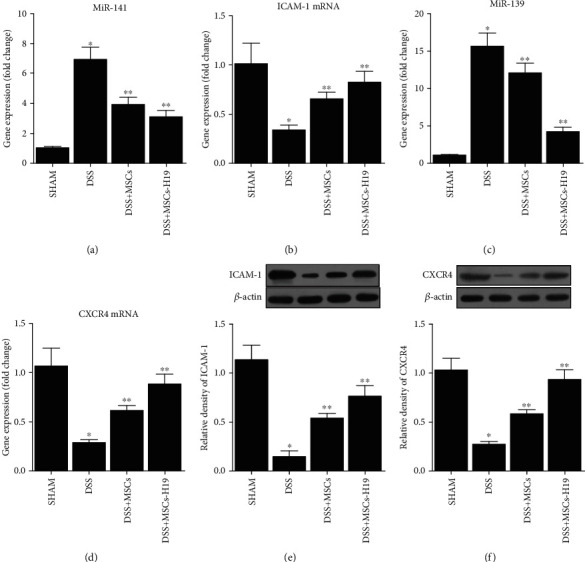
Effects of DSS, MSC, and MSC-H19 on miR-141, ICAM-1 mRNA, miR-139, and CXCR4 levels (^∗^*P* value <0.05 vs. control; ^∗∗^*P* value <0.05 vs. DSS). (a) RNA level of miR-141 in mice was the highest in the DSS group and the lowest in the SHAM group. (b) RNA level of ICAM-1 in mice was the highest and lowest, respectively, in the SHAM and DSS groups. (c) RNA level of miR-139 in mice was the highest in the DSS group and the lowest in the SHAM group. (d) RNA level of CXCR4 in mice was the highest and lowest, respectively, in the SHAM and DSS groups. (e) Protein level of ICAM-1 in mice was the highest and lowest, respectively, in the SHAM and DSS groups. (f) Protein level of CRCX4 in mice was the highest and lowest, respectively, in the SHAM and DSS groups.

**Figure 8:**
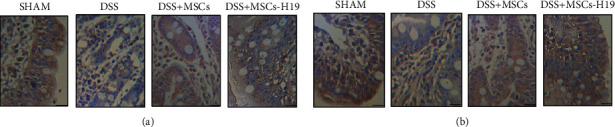
Protein levels of ICAM-1 and CXCR4 in transverse colon tissues of mice in SHAM, DSS, DSS + MSC, and DSS + MSC-H19 groups were detected by IHC (the brown color denotes the expression of ICAM-1 in (a) and CXCR4 in (b); scale bar: 50 *μ*m). (a) IHC assay indicated that the protein level of ICAM-1 was the lowest in the DSS group and highest in the SHAM group. (b) IHC assay indicated that the protein level of CXCR4 was the lowest in the DSS group and highest in the SHAM group.

We further tested the targets of H19 (i.e., miR-141 and miR-139) and their downstream genes (i.e., ICAM-1 and CXCR4) in the four mouse groups. As shown in Figure 7, administration of DSS evidently increased miR-141 (Figure 7(a)) and miR-139 (Figure 7(c)) expression while decreasing ICAM-1 mRNA (Figure 7(b)) and CXCR4 mRNA (Figure 7(d)) expression, which was also confirmed by RT-qPCR. MSC injection weakened the effects of DSS, and MSC-H19 further abated the effects of DSS. ICAM-1 and CXCR4 protein expression was measured via Western blotting. As shown in Figures 7(e) and 7(f), the protein expression of ICAM-1 and CXCR4 was significantly decreased by DSS, while MSC infusion elevated the protein expression of CXCR4 and ICAM-1, and MSC-H19 had a more prominent effect in raising the ICAM-1 and CXCR4 expression (Figures 7(e) and 7(f)). The protein expression of CXCR4 (Figure 8(b)) and ICAM-1 (Figure 8(a)) was further examined via IHC, and the results were consistent with the WB results.

## 5. Discussion

IBD, such as Crohn's disease and ulcerative colitis, is persistent and relapsing conditions with chronic inflammation of the intestinal tract and injuries to the mucus [31]. Mucosal recovery is linked to improved and lasting medical efficacy. Thus, mucosal recovery has been a major target for IBD treatment [32]. The absence of mucosal recuperation generally leads to difficulties in patient recovery and complications, such as blood loss, fistulas, and perforation, which demand a hospital stay and surgery. Less than 50% of IBD patients can be managed with standard treatments, such as immunomodulators, corticosteroids, and biologic drugs [33]. Standard treatments used to treat IBD rely on suppression of the immune system; for that reason, new techniques are required to enhance mucosal regrowth [34]. While intravenous transplants of stem cells are frequently used in common treatments, the distribution of MSC to wounded tissues is still extremely complicated [35]. Initiatives are being made to promote the delivery of MSC, such as by using nanoparticles [36]. In addition, chemokine CXC receptor (CXCR), vascular cell adhesion molecule 1 (VCAM1), and E-selectin ligands have been assessed to cover MSC surfaces to aid their targeted delivery to wounded sites, improving the therapeutic results of IBD therapy [35, 37]. In the present study, mice were randomly divided into 4 groups: SHAM, DSS, DSS + MSC, and DSS + MSC-H19. Mice treated with DSS showed significant weight loss and shortened colon length. The effects of DSS were obstructed by MSC treatment and were further alleviated by MSC-H19 treatment. In addition, the level of inflammatory cytokines was tested in the four groups. DSS treatment induced an inflammatory response, while MSC injection reduced the effects of DSS, and MSC-H19 infusion further reduced the inflammatory response. Furthermore, the levels of targets of H19 (miR-141 and miR-139) and their downstream genes (ICAM-1 and CXCR4) were tested in the four mouse groups. It was found that the expression of miR-141 and miR-139 was evidently increased while the expression of ICAM-1 and CXCR4 was decreased in DSS animals. MSC injection weakened the effects of DSS, and MSC-H19 injection further abated the effects of DSS.

Recently, the level of H19 was discovered to become substantially enhanced in osteoarthritis tissues, indicating a prospective role of H19 in the progression of inflammatory conditions [38]. A previous report on the role of H19 in protection of the intestinal barrier in sepsis seems to conflict with our results [39]. Such a discrepancy could be attributed to the different disease backgrounds. The basic disease investigated in this study is ulcerative colitis, and the basic disease examined in the reference is sepsis. Recently, scientists utilized bioinformatic and luciferase assay to verify the binding between miR-141 and H19. As assumed, it was found that miR-141 could bind complementarily to H19, inhibiting translation of an RLuc-H19 gene. It was likewise revealed that miR-141 acts as a repressor of ICAM-1 expression, and the miR-141 overexpression decreases the ICAM-1 expression induced by ischemia to alleviate reperfusion injury. Therefore, miR-141 might act as a beneficial factor in the treatment of heart disease [40]. Here, ICAM-1 is predicted to be a downstream gene of miR-141, and CXCR4 is a downstream target of miR-139. The activity of miR-141/miR-139 was decreased in cells cotransfected with miR-141/miR-139 and ICAM-1/CXCR4. In addition, mesenchymal stem cells (MSC) were transfected with pcDNA, pcDNA-H19, or H19 siRNA. The expression levels of miR-141 and miR-139 were decreased in pcDNA-H19 cells and increased in H19 siRNA cells, while the levels of ICAM-1 and CXCR4 were increased in pcDNA-H19 cells but decreased in H19 siRNA cells. Furthermore, MSC were transfected with pcDNA, scramble control, pcDNA-H19, or H19 siRNA. MTT and transwell assays revealed that the higher H19 expression had positive effects on cell proliferation and migration, while downregulation of H19 inhibited cell proliferation and migration.

ICAM-1 has been shown to participate in the vital processes of immune reactions [9]. ICAM-1 can promote homing of numerous immune cells in secondary lymphoid organs. From a physiological standpoint, the ICAM-1 expression in MSC is very low, but ICAM-1 is dramatically enhanced in MSC located in an inflammatory environment [41, 42]. In addition, ICAM-1 u-regulation enhances the immunosuppressive role of MSC [41–43].

The expression of ICAM-1 in IBD indicates a possible function of ICAM-1 in IBD pathophysiology. ICAM-1 was shown to be elevated in colon lysates collected from patients with ulcerative colitis [44, 45].

The level of CXCR4 is an essential factor in promoting the proliferation and migration of stem cells. CXCR4 is mostly expressed in cell cytoplasm [46]. The level of CXCR4 is minimized with extended cell culture [47]. Various cytokines can promote the expression of CXCR4 on the cell membrane [48, 49]. Transforming growth factor-*β*1 (TGF-*β*1) might upregulate the CXCR4 expression in basal cell carcinoma (BCC) cells [50]. TGF-*β*1 can also increase CXCR4 levels in MSC in acute I/R injury. Additionally, CXCR4 aids MSC migration to SDF-1.

The results of this study provided further understanding of the molecular mechanism underlying the therapeutic effect of stem cell in the treatment of IBD and identified the reinforcer, H19, to improve the therapeutic effect of stem cells. These findings may provide a basis for the future clinical use of stem cell as a modality to treat IBD.

## 6. Conclusion

In conclusion, collectively, the results of our study demonstrated that the overexpression of H19 in MSC downregulated the expression of miR-139 and miR-141, thus upregulating the expression of their target genes ICAM-1 and CXCR4, respectively. Since it has been verified that the overexpression of ICAM-1 and CXCR4 can promote the homing of MSC in the treatment of ulcerative colitis, the overexpression of H19 exhibited therapeutic effects in ulcerative colitis.

## Data Availability

The data of this study are available from the corresponding author upon reasonable request.
